# Minocycline intra-bacterial pharmacokinetic hysteresis as a basis for pharmacologic memory and a backbone for once-a-week pan-tuberculosis therapy

**DOI:** 10.3389/fphar.2022.1024608

**Published:** 2022-10-18

**Authors:** Devyani Deshpande, Shashikant Srivastava, Jotam Garaimunashe Pasipanodya, Tawanda Gumbo

**Affiliations:** ^1^ Baylor University Medical Center, Dallas, TX, United States; ^2^ Department of Pulmonary Immunology, University of Texas Health Science Center at Tyler, Tyler, TX, United States; ^3^ Quantitative Preclinical and Clinical Sciences Department, Praedicare Inc, Dallas, TX, United States; ^4^ Hollow Fiber System and Experimental Therapeutics Laboratories, Praedicare Inc., Dallas, TX, United States

**Keywords:** post antibiotic microbial killing, gama slope, hollow fiber model system, post-antibiotic effect, shannon entropy

## Abstract

**Background:** There is need for shorter duration regimens for the treatment of *tuberculosis*, that can treat patients regardless of multidrug resistance status (pan-tuberculosis).

**Methods:** We combined minocycline with tedizolid, moxifloxacin, and rifampin, in the hollow fiber system model of *tuberculosis* and mimicked each drugs’ intrapulmonary pharmacokinetics for 28 days. Minocycline-tedizolid was administered either as a once-a-week or a daily regimen. In order to explore a possible explanation for effectiveness of the once-a-week regimen, we measured systemic and intra-bacterial minocycline pharmacokinetics. Standard daily therapy (rifampin, isoniazid, pyrazinamide) was the comparator. We then calculated *γ*_f_ or kill slopes for each regimen and ranked the regimens by time-to-extinction predicted in patients.

**Results:** The steepest *γ*_f_ and shortest time-to-extinction of entire bacterial population was with daily minocycline-rifampin combination. There was no difference in *γ*_f_ between the minocycline-tedizolid once-a-week *versus* the daily therapy (*p* = 0.85). Standard therapy was predicted to cure 88% of patients, while minocycline-rifampin would cure 98% of patients. Minocycline concentrations fell below minimum inhibitory concentration after 2 days of once-weekly dosing schedule. The shape of minocycline intra-bacterial concentration-time curve differed from the extracellular pharmacokinetic system and lagged by several days, consistent with system hysteresis. Hysteresis explained the persistent microbial killing after hollow fiber system model of *tuberculosis* concentrations dropped below the minimum inhibitory concentration.

**Conclusion:** Minocycline could form a backbone of a shorter duration once-a-week pan-tuberculosis regimen. We propose a new concept of post-antibiotic microbial killing, distinct from post-antibiotic effect. We propose system hysteresis as the basis for the novel concept of pharmacologic memory, which allows intermittent dosing.

## Introduction

The scourge of *tuberculosis* (TB) has been exacerbated by the emergence of multidrug-resistant TB (MDR-TB) and extensively drug-resistant TB (XDR-TB) (Dheda et al., 2017). Therapy for MDR-TB has a success rate of about 50%, while that for XDR-TB is about 20%, and patients with XDR-TB often die within 10-month (Dheda et al., 2017). The recently approved regimen of bedaquiline, pretomanid and linezolid cures greater than 85% of patients with drug-resistant TB when therapy is administered over 6-month (WHO, 2022). However, the adverse side-effect profile is high such that this combination regimen is recommended for highly resistant TB with no other treatment option (Conradie et al., 2020). Therefore, there is an urgent need to find drugs that could be immediately used to treat these unfortunate patients safely and effectively.

There are several possible approaches to improve the therapeutic outcomes of the treatment regimens, including 1) repurposing old drugs used for other indications and found to be effective against *Mycobacterium tuberculosis* (*Mtb*), and 2) develop new specific anti-TB drugs that are safe and effective, among others. While both strategies are commonly used; the latter strategy requires several years to decades before a drug and resultant combination regimen(s) can be used in the clinic. With the repurposing strategy, β-lactam/β-lactamase inhibitors, minocycline and its congener tigecycline, and new oxazolidinones such as tedizolid, have been found to be highly effective anti-TB drugs (Ramon-Garcia et al., 2016; Deshpande et al., 2017; Deshpande et al., 2018b; Srivastava et al., 2018b; Deshpande et al., 2019a; Deshpande et al., 2019b; Srivastava et al., 2020b; Srivastava et al., 2021a; Srivastava et al., 2021b). Minocycline kills both extracellular and intracellular bacilli directly, and the optimal dose for MDR-TB was identified as 7 mg/kg (Deshpande et al., 2019b). In addition, minocycline demonstrated dose-dependent anti-inflammatory activity, including inhibition of sonic hedgehog-patched-gli signaling, which has implications for improving lung remodeling (Deshpande et al., 2019b). Here, we performed pharmacokinetics-pharmacodynamics (PK/PD) studies using the hollow fiber system model of TB (HFS-TB) to determine if minocycline can be used as the backbone of the once-a-week combination regimen (Alffenaar et al., 2020). Microbial kill rates with different experimental regimens were calculated using $\gamma_{f}$ slopes (Gumbo et al., 2004; Magombedze et al., 2018; Srivastava et al., 2019; Magombedze et al., 2021; Gumbo et al., 2022).

## Methods

### Materials and bacterial strains

*Mtb* reference laboratory strains, the virulent strain and attenuated virulence strain that were cultured from the *Mtb* H37 parent strain isolated from a patient in 1905, and designated Mtb H37Ra (ATCC#25177) and H37Rv (ATCC# 27294), were used in the studies (Steenken and Gardner, 1946). *Mtb* were grown to logarithmic phase (log-phase) growth in Middlebrook 7H9 broth plus 10% oleic acid, albumin, dextrose, and catalase (OADC) (herein called “broth”) under 5% CO_2_ at 37°C for 4 days before each experiment. All study drugs were purchased from Sigma-Aldrich except moxifloxacin, which was purchased from the hospital pharmacy. Hollow fiber cartridges were obtained from FiberCell systems. Mycobacterial Growth Indicator Tube (MGIT) liquid culture systems and supplies were purchased from Becton Dickinson.

### Comparison of combination regimens in the hollow fiber system model of *tuberculosis*

The HFS-TB has been described in detail in the past in a number of our previous publications (Deshpande et al., 2017; Deshpande et al., 2018b; Srivastava et al., 2018b; Deshpande et al., 2019a; Deshpande et al., 2019b; Srivastava et al., 2020b; Srivastava et al., 2021a; Srivastava et al., 2021b). We utilized log-phase growth extracellular bacilli in the HFS-TB studies instead of intracellular bacilli to eliminate the prolonged persistence of drugs inside infected macrophages and to eliminate the pro-apoptotic effect of minocycline which we have shown elsewhere as a mechanism of *Mtb* kill (Deshpande et al., 2019b). There were two HFS-TB studies performed.

The first HFS-TB study was performed to determine if the different combinations of minocycline could be used as a backbone of a once-a-week regimen. We inoculated 20 ml log-phase growth *Mtb* H37Ra cultures into the peripheral compartment of the HFS-TB units, after which the systems were treated once daily with one of several experimental regimens over a 28-day study period. There were two HFS-TB replicates per regimen as follows: 1) isoniazid (300 mg/day) plus rifampin (600 mg/day), 2) isoniazid plus rifampin plus pyrazinamide (1.5 g/day) [standard regimen], 3) minocycline (7 mg/kg/day) plus rifampin, 4), minocycline plus moxifloxacin (800 mg/day), 5) minocycline plus tedizolid (200 mg/day), 6) minocycline plus tedizolid once-a-week, 7) non-treated controls. We utilized intrapulmonary pharmacokinetics (PKs) of minocycline and tedizolid at a half-life of 13 h, and a pulmonary-to-serum free drug AUC ratio of 3.8 for minocycline and 4.0 for tedizolid (Naline et al., 1991; Housman et al., 2012; Flanagan S. D. et al., 2014). Minocycline was administered at a weekly (168 h) AUC/MIC ratio of 440 and tedizolid at 1,800. Isoniazid and rifampin doses were at a 3 h half-life, pyrazinamide half-life was set to 12 h, and moxifloxacin half-life at 6 h (Gumbo et al., 2004; Srivastava et al., 2011). The central compartment of each HFS-TB unit was sampled at pre-determined timepoints for drug concentration measurements, whereas the peripheral compartment was sampled for enumeration of the bacterial burden either using the solid agar (Middlebrook 7H10 supplemented with 10% OADC) culture method or by inoculating the MGIT tubes to record the time-to-positive (TTP) as the second pharmacodynamic measure.

The second HFS-TB study was performed to identify intra-bacterial concentrations (henceforth shortened to “bacterial PKs”) of minocycline *versus* microbial kill using *Mtb* H37Rv to explain prolonged bacterial kill with minocycline. After inoculation of 20 ml of bacterial cultures, minocycline and tedizolid were administered as a single bolus at t = 0. Simultaneous central compartment and peripheral compartment sampling were performed at 0 h (pre-dose) followed by 1, 6, 24, 48, 72, 96, 120, 144 and 168 h post-dosing for measurement of extracellular drug concentration as well as bacterial PKs. For the intra-bacterial drug concentration measurement, 1 mL sample from the peripheral compartment was added to dolphin tubes pre-filled with silicone oil mixture, as we have described elsewhere, at a ratio of 1:1 (Gumbo et al., 2007). Centrifugation was performed at 13,000 rpm for 5 min following which the bacterial pellet was collected in 70% acetone. Additionally, to ensure there was no degradation of drugs from acetone, we spiked three sets of samples with a known amount of three different minocycline concentrations and added either 70% acetone or broth, to which we blinded the team of researchers responsible to measure the drug concentrations. Acetone was allowed to evaporate prior to measurement of intra-bacillary drug concentrations using the assay described in our previous publications (Srivastava et al., 2011; Srivastava et al., 2018b; Deshpande et al., 2019b). The samples from the peripheral compartment of each HFS-TB unit were also inoculated into MGIT tubes to record the TTP as the second pharmacodynamic measure.

### Pharmacokinetic analyses

Compartmental PK analyses of drug concentrations were performed using ADAPT software from Biomedical Simulations Resource (BMSR) at the University of Southern California (D'Argenio et al., 2009). For the concentration of minocycline inside *Mtb*, we assumed an *Mtb bacillus* volume of 8.4 µm^3^ based on the bionumbers details - (http://bionumbers.hms.harvard.edu/bionumber.aspx?id=101691). The bacterial burden of *Mtb* at each sampling point was factored in while calculating the total bacterial volume at each time point.

### Pharmacodynamic analyses using γ-slopes

Previously we have published a system of ordinary differential Eqs. 1,2, that were applied to both patients sputa and the HFS-TB readouts, allowing us to map back-and-forth between HFS-TB and patients using morphisms and extinction mathematics (Gumbo et al., 2004; Magombedze et al., 2018; Srivastava et al., 2019; Magombedze et al., 2021). The equations are:

$$\frac{dB_{f}}{dt}=\left({1-\epsilon }_{f}\right)r_{f}B_{f}\left(1-\frac{B_{s}+B_{f}}{K_{\max}}\right)-\gamma_{f}B_{f},$$ (1)

$$\frac{dB_{s}}{dt}=\left({1-\epsilon }_{s}\right)r_{s}B_{s}\left(1-\frac{B_{s}+B_{f}}{K_{\max}}\right)-\gamma_{s}B_{s}.$$ (2)

where, $B_{f}$ is CFU/mL of *fast* replicating bacteria, $B_{s}$ is semidormant/non-replicating [*slow*] bacteria CFU/mL, t is time, r_f_ and r_s_ model the rate of replication of the fast and slow replicating bacteria, $K_{\max}$ is bacteria-carrying capacity, $\gamma_{s}$ and $\gamma_{f}$ are the antibiotic regimen kill slopes for slow and fast replicating bacteria respectively. Here, we used fast replicating bacteria in the HFS-TB, and thus applied Eq. 1 for comparisons of regimens. All model parameter estimates were calculated directly from HFS-TB CFU/mL and TTP *versus* time data; none were fixed based on prior work. We then mapped these to patients, using the translation factor derived and described elsewhere (Magombedze et al., 2018).

### Monte-Carlo experiments (MCE) for dose selection to use in once-a-week regimen

Since PK variability and resultant drug concentrations, drug penetration into lung lesions, PK/PD parameters, and MICs explain most of the variance in therapy outcomes in TB patients, modeling for dose selection should take into account PK and MIC variability (Pasipanodya et al., 2013; Gumbo et al., 2014a; Gumbo et al., 2014b; Chigutsa et al., 2015; Deshpande et al., 2018a; Dheda et al., 2018). We performed MCE using ADAPT 5 software, with steps detailed in the past, to identify the once-a-week minocycline and tedizolid dose that would achieve the PK/PD exposures achieved by each of these drugs in the dual therapy regimen (Gumbo et al., 2004; Pasipanodya and Gumbo, 2011). For the domain of input, we utilized the minocycline PK parameter estimates from the MINOS study in which patients received a dose of 10 mg/kg daily, and from two other separate studies that also identified similar PK parameters but at lower doses (Welling et al., 1975; Yamamoto et al., 1999; Fagan et al., 2010). For minocycline, we assumed an oral absorption of 100%, and a lung-to-serum penetration ratio of 3.8 based on prior studies (Naline et al., 1991). The MIC distribution used was that we identified with clinical strains in the past (Deshpande et al., 2019b). For tedizolid, we used PK parameter estimates from the study of Flanagan et al., and a free drug lung-serum AUC ratio of 4 (Housman et al., 2012; Flanagan S. et al., 2014; Srivastava et al., 2018b). We used the tedizolid MIC distribution identified by Vera-Cabrera *et al* (Vera-Cabrera et al., 2006). We examined the tedizolid doses of 350, 700 mg, 1,000 mg, and 1,400 mg administered as a single dose once a week. Target exposures were those achieved in the HFS-TB shown in Table 1.

**TABLE 1:** Drug exposures of antibiotics achieved in the HFS-TB units.

	Cumulative weekly AUC_0-168_ [mg*h/L]	AUC_0-24_ [mg*h/L]	AUC_0-24_/MIC
Minocycline daily	204.2	29.14	58.28
Minocycline once weekly	219	31.29	62.58
Tedizolid daily	1,353	193.3	386.6
Tedizolid once weekly	904.4	129.2	258.4
Isoniazid daily	-	32.43	518.88
Rifampin daily	-	10.04	160.64
Moxifloxacin daily	-	108.3	1,442.4
Pyrazinamide daily	-	1,371	54.84

## Results

### Minimum inhibitory concentration

The MIC of drugs against H37Rv were as following: isoniazid 0.06 mg/L, rifampin 0.125 mg/L, pyrazinamide 12.5 mg/L, minocycline 2 mg/L, moxifloxacin 0.25 mg/L, and tedizolid 0.25 mg/L, similar to those reported in prior publications (Srivastava et al., 2011; Srivastava et al., 2020a). The MICs against H37Ra were as following: isoniazid 0.06 mg/L, rifampin 0.06 mg/L, pyrazinamide 25 mg/L, minocycline 0.5 mg/L, moxifloxacin 0.125 mg/L, and tedizolid 0.25 mg/L, as has been reported in our prior publications (Srivastava et al., 2018a; Deshpande et al., 2018c; Deshpande et al., 2019b).

### Hollow fiber system model of *tuberculosis* results

**FIGURE 1:**
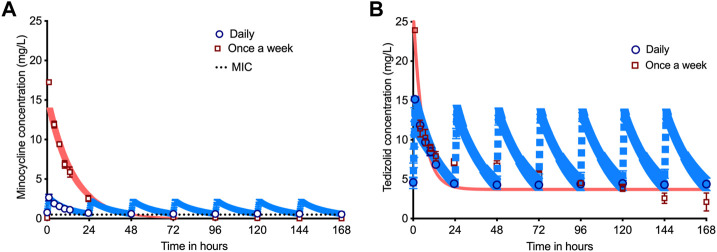
Pharmacokinetics of minocycline and tedizolid in dual therapy. The symbols are mean concentrations, error bars are standard deviation, and shaded areas are pharmacokinetic (PK) model predicted concentrations. The blue line is PK-model predicted for daily therapy, while the salmon colored line is PK-model predicted for once a week therapy. **(A)** Concentration-time profile for the once-a-week minocycline doses compared to daily doses demonstrated that minocycline concentration declined below the MIC by 48 h (28.57% of the once-a-week dosing interval) for the once-a-week dosing schedule but stayed above the MIC for 100% of 168 h with daily dose. **(B)** Concentration-time profile for the companion once-a-week tedizolid doses *versus* daily dose demonstrates that the tedizolid peak concentration could not reach 7 times that of the daily dose due to solubility issues, which means that the once-a-week regimen was prejudiced compared to daily therapy since the daily therapy schedule achieved a 49.6% higher cumulative AUC per week than once-a-week regimen.

Figure 1A and Supplementary Figure S1 show the concentration-time profile of drugs achieved in the HFS-TB, with Table 1 listing the AUCs achieved by each of the drug based on concentration measurements in the HFS-TB units. Table 2 compares the intended *versus* achieved peak concentrations (C_max_) in the HFS-TB and demonstrates the accuracy of HFS-TB in achieving the intended drug concentrations. The C_max_ and AUCs were in the range achieved by standard dose rifampin and isoniazid, and high dose pyrazinamide and moxifloxacin, inside TB lesions (Pasipanodya et al., 2013; Dheda et al., 2018; Ordonez et al., 2020). Minocycline concentrations in the HFS-TB fell below MIC after 48 h of drug administration when given as a once-a-week regimen. To reiterate, the minocycline concentrations in the HFS-TB treated with the once-a-week schedule were below the MIC between day 3–7 (with once-a-week dosing schedule).

**TABLE 2:** Intended *versus* HFS-TB measured peak concentration of each drug in the regimen.

Drug	Intended C_max_ (mg/L)	Measured C_max_ (mg/L)
Isoniazid	6.8	4.75 ± 0.29
Rifampin	3	1.88 ± 0.45
Pyrazinamide	54	83.31 ± 6.07
Moxifloxacin	8.4	7.28 ± 0.79
Minocycline	2.6	2.67 ± 0.41
Tedizolid	2.6	2.37 ± 0.64

**FIGURE 2:**
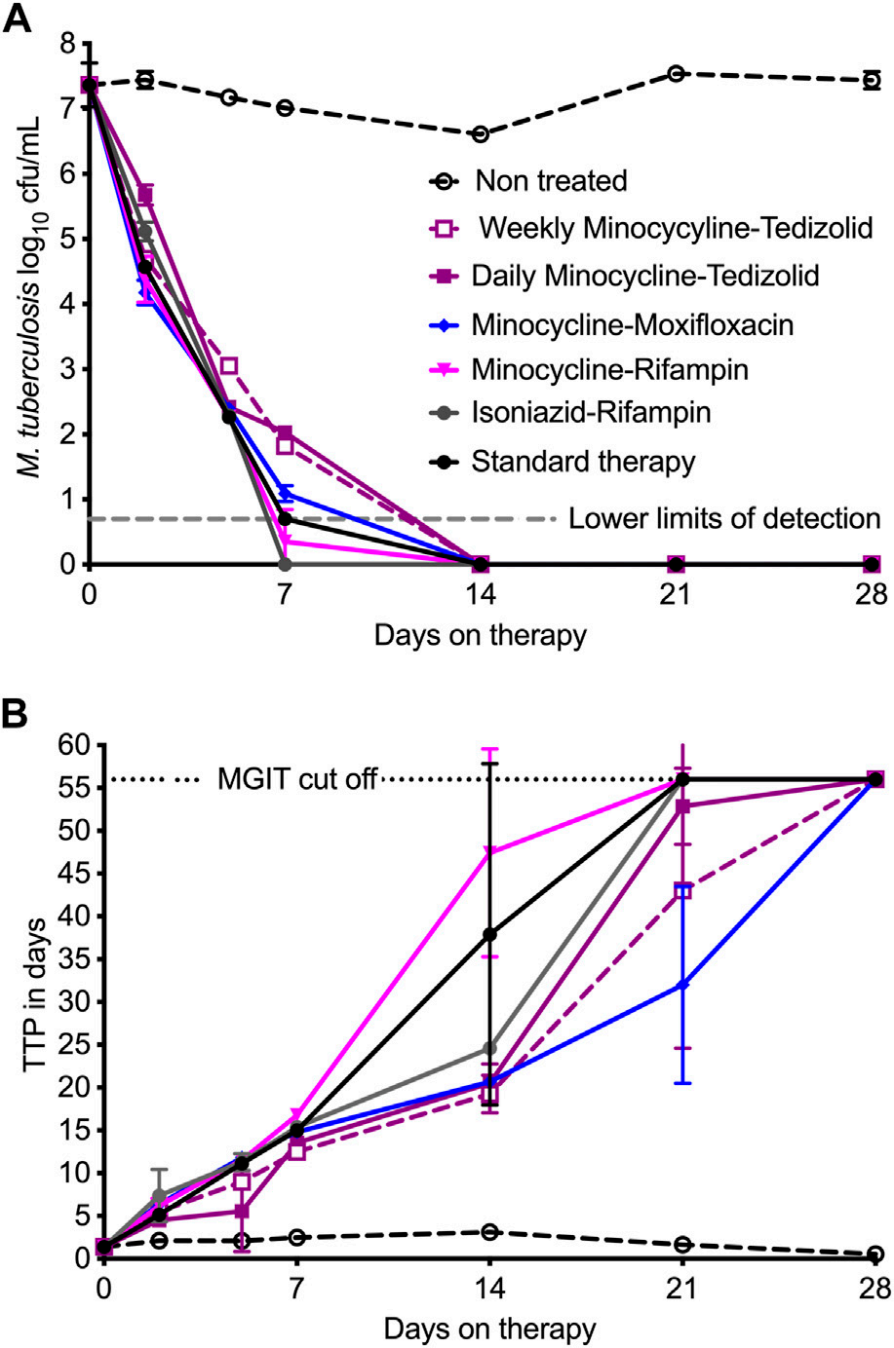
Comparisons of dual therapy regimens to standard triple therapy. Symbols are mean and error bars are standard deviation. **(A)** Using cfu/mL readouts, minocycline-rifampin and isoniazid-rifampin fell below the lower limits of detection by day 7, and by this metric would be the highest ranked. The kill pattern was not necessarily exponential decline and differed between the regimens. **(B)** TTP, with negative culture call at 56 days, had a wider dynamic range and shows that cultures only became negative on day 21 for all regimens, except for minocycline-tedizolid daily and once-a-week regimens which achieved that on day 28.

Figure 2A shows the time-kill curves for log_10_ cfu/mL, which is the traditional method of bacterial burden quantification that allowed us to compare results presented here with our previous HFS-TB studies. Minocycline-rifampin and rifampin-isoniazid regimen treated HFS-TB units had negative cultures by day 7, whereas all other regimens showed negative cultures by the study day 14. Figure 2B shows the time-kill curves by each regimen in the HFS-TB, using MGIT derived TTP readouts; the lower the bacterial burden the higher the TTP. In our MGIT assay, the time-in protocol was set to 56 days (compared to 42-day used in the clinical microbiology laboratories) after which the samples were recorded as negative for bacterial growth. Based on TTP readout, therapy duration (time) to negative cultures was 21 days for minocycline-rifampin, isoniazid-rifampin, and standard therapy, while remaining regimens took one more week to achieve negative culture.

### Regimen ranking using γ-slopes

**FIGURE 3:**
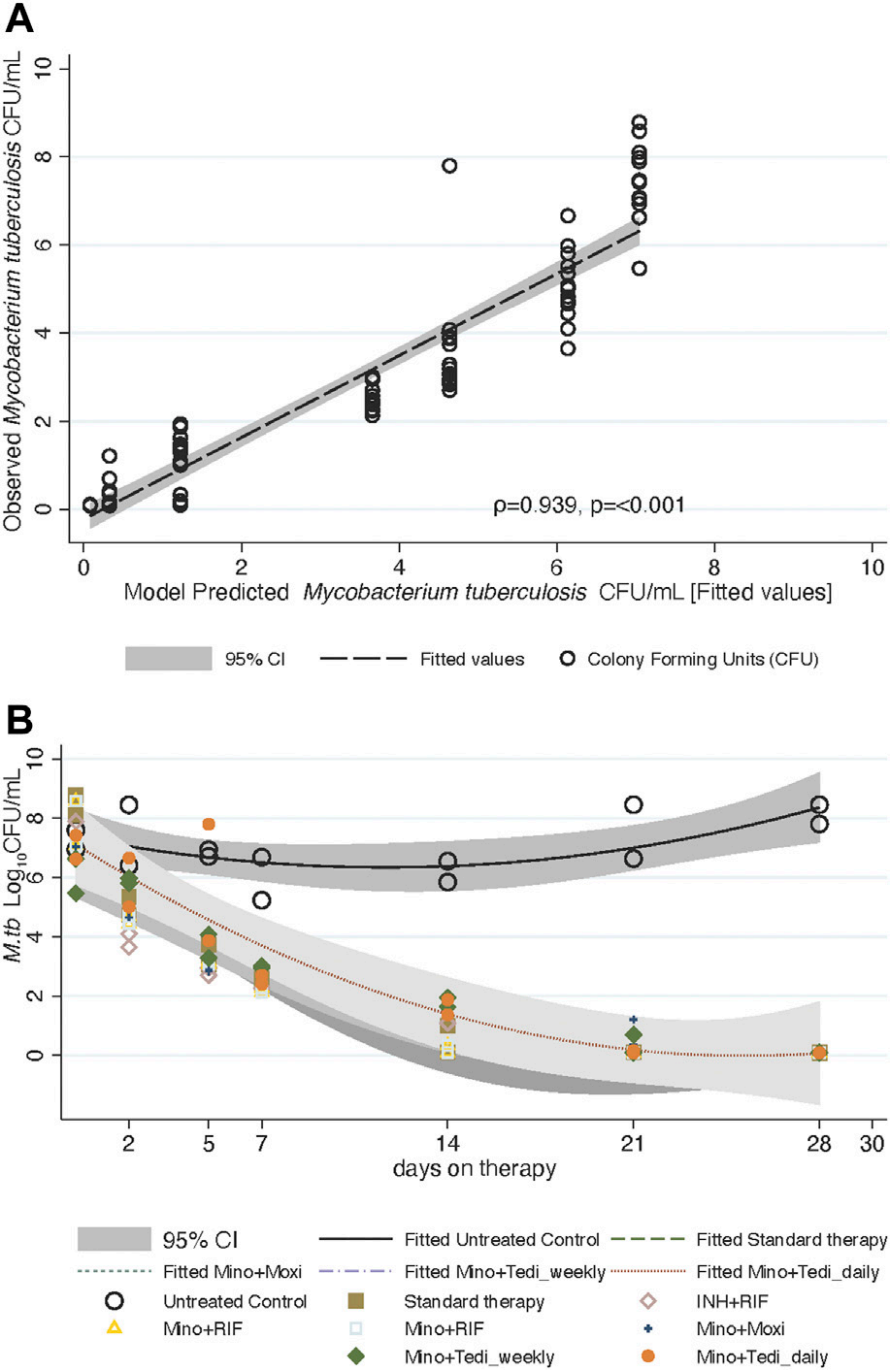
*Mycobacterium tuberculosis* trajectories and $\gamma_{f}$ in HFS-TB treated. **(A)** The modeled cfu/mL *versus* observed cfu/mL using ordinary differential equation ^#^1 for all data points are shown, as a diagnostic. The model was a very good fit. **(B)** Shaded areas are 95% credible intervals. Many of the $\gamma_{f}$ overlapped, but the most steep (deepest gray) was the minocycline-rifampin combination regimen. In the non-treated HFS-TB, bacteria grew.

The use of γ-slopes offers the advantage of integrating both cfu/mL values and TTP readouts into one equation, and the approach is agnostic of kill pattern, and thus can be used to rank regimens by $\gamma_{f}$ (speed of kill), or time-to-extinction (shortest duration of therapy), or proportion of patients expected to be cured for all time points (Gumbo et al., 2022). The $\gamma_{f}$ slopes of each regimen in the HFS-TB are shown in Figure 3 and Table 3. We utilize standard therapy outcomes for model validation and quality control and Table 2 shows that the $\gamma_{f}$ and time-to-extinction was similar to that observed in the HFS-TB in the past (Magombedze et al., 2018), and the prediction that 88% (95% credible intervals: 80%–94%) of patients would be cured at all time points is virtually identical to clinical observations. This means that both the HFS-TB experiments and the modeling performed according to specifications and standard operating procedures. Figure 3 and Table 3 show that the minocycline-rifampin combination regimen was ranked top by $\gamma_{f}$ , time-to-extinction, and the predicted proportion of patients cured at all time points. Moreover, the $\gamma_{f}$ of the daily and once-a-week minocycline-tedizolid combination overlapped (*p* = 0.85), as did time-to-extinction and proportion of patients cured. In other words, the pharmacodynamic effects of daily therapy and once a week therapy with minocycline-tedizolid were similar. Since the minocycline-tedizolid combination had the lowest $\gamma_{f}$ of all (slowest kill speed), but the once-a-week regimen was as good as the daily, it meant that the once-a-week regimen must continue to kill *Mtb* long after the drug concentrations fell below the MIC.

**TABLE 3:** ODE derived parameters of *γ*_f_ and time-to-extinction in the HFS-TB, and proportion of patients expected to be cured.

Rank	Regimen	Data points	Mean negative *γ*_f_ log_10_ cfu/ml/day (95% CI)	Median time-to-extinction in days (95% CrI)	Mean proportion cured all time points (95%CI)
1	Minocycline-rifampin*^/^**	14	0.34 (0.25–0.43)	20.24 (10.68–70.76)	0.98 (0.93–1.00)
2	Standard Therapy*	14	0.34 (0.25–0.44)	19 (4.36–105)	0.88 (0.80–0.94)
3	Minocycline-moxifloxacin	14	0.26 (0.17–0.34)	62.94 (22.33–483.07)	0.53 (0.43–0.63)
4	Isoniazid-rifampin	14	0.25 (0.15–0.35)	29.18 (12.78–148.99)	0.92 (0.84–0.96)
5	Minocycline-tedizolid daily	14	0.20 (0.10–0.30)	29.96 (13.59–156.89)	0.82 (0.73–0.89)
5	Minocycline-tedizolid weekly	14	0.19 (0.13–0.21)	43.39 (25.44–129.81)	100

*p = 0.038 compared to minocycline-tedizolid daily and **p = 0.003 compared to minocycline-tedizolid weekly.

### Minocycline intra-bacterial PKs and pharmacologic memory

**FIGURE 4:**
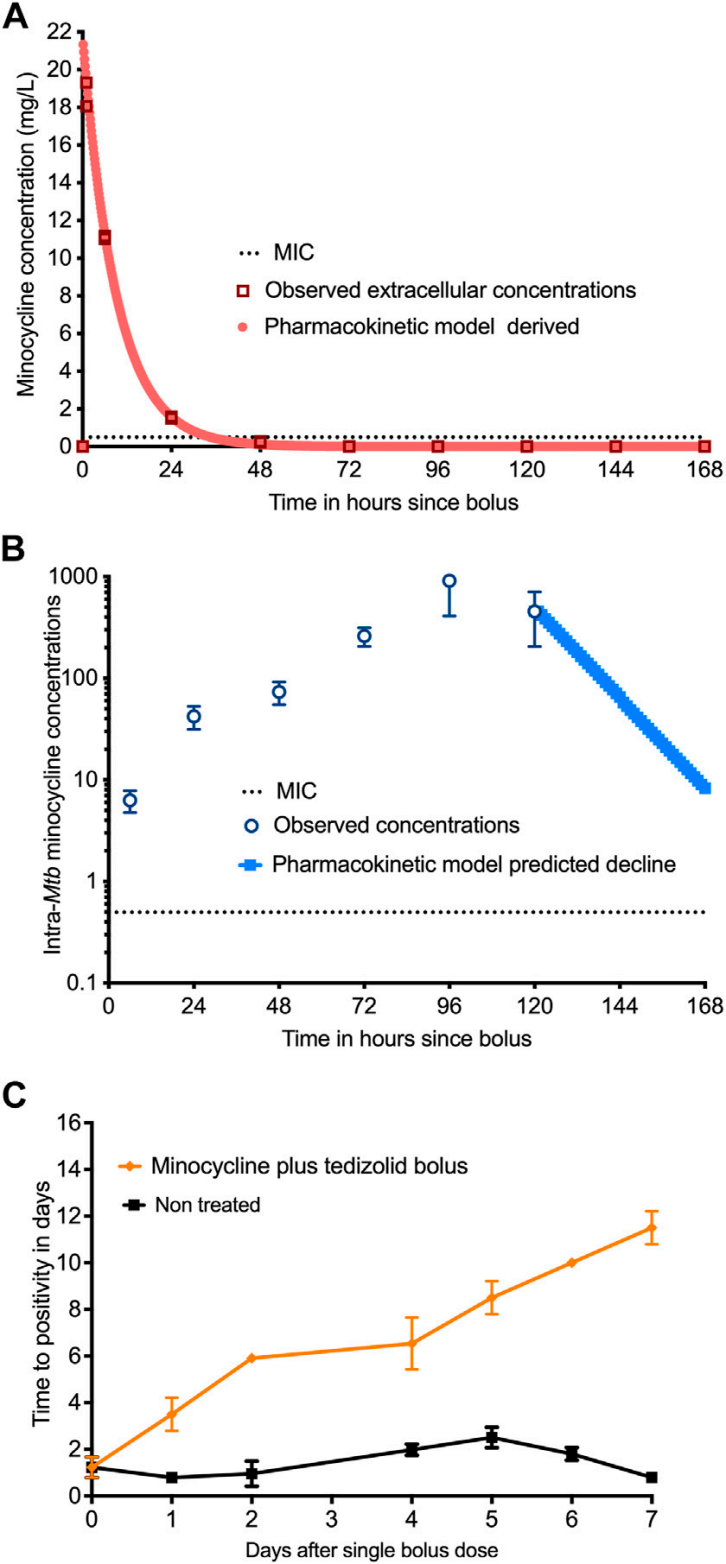
Measurement of intra-bacterial minocycline concentrations. **(A)** Extracellular minocycline concentrations following once-a-week dosing against *Mtb* fell below MIC after 48 h similar to the first HFS-TB experiment. **(B)** Intra-bacterial (inside *Mtb*) minocycline concentrations persisted above MIC well beyond 48 h, and the concentration-time curve lagged by 4 days behind extra-bacterial pharmacokinetics. **(C)** There were three replicate HFS-TB units treated with a single bolus dose of tedizolid and minocycline, at a half-life of 12 h. In parallel with measuring the intra-bacterial concentrations, we also quantified the bacterial burden using TTP in days. The figure shows continued microbial kill even after the minocycline extracellular concentrations dropped below the MIC in extracellular HFS-TB compartment by 48 h, which was however, parallel to the intra-*Mtb* concentrations.

To understand why minocycline continues to kill 4 days after drug concentrations declined below the MIC in the HFS-TB, we investigated the possibility of longer persistence of drug inside bacteria as a possible explanation. We treated log-phase growth *Mtb* H37Rv with a single bolus of minocycline and tedizolid, in triplicate HFS-TB, at deliberately shorter half-life for minocycline, and measured HFS-TB external compartment concentrations daily over 7 days. Figure 4A shows the results of the model derived *versus* measured extracellular minocycline concentrations in the HFS-TB. The minocycline clearance rate in the HFS-TB was 0.035 ± 0 L/h with a volume of 0.320 ± 0.004 L and a half-life of 6.34 ± 0.852 h. The minocycline bacterial PKs were those shown in Figure 4B. Figure 4B shows that while there was a bolus given to the HFS-TB the time to peak concentration inside *Mtb* was 96 h, followed by a decline, such that the shape of the minocycline concentration-versus-time decline inside *Mtb* was different from that in the extracellular HFS-TB. This is consistent with system hysteresis (Ewing, 1882; Mayergoyz, 2003), which led to minocycline concentrations persisting inside *Mtb* for at least 4-day after the drug concentration declined below limits of detection in extracellular fluid in the HFS-TB. The mass-charge ratio, by the LC-MS/MS assay, on each day demonstrated that it was the intact minocycline molecule that persisted inside the *Mtb* and not its metabolites. Figure 4C shows the corresponding TTPs during the 7-day HFS-TB study. The TTP in non-treated controls stayed relatively constant in the 7 days Figure 4C demonstrated continued microbial kill beyond day 2 (i.e, progressively increasing TTPs), during the time period of 2–7 days when minocycline concentration persisted inside the bacteria but had fallen below detection in the HFS-TB extracellular fluid.

### Target attainment probability with clinical doses

**FIGURE 5:**
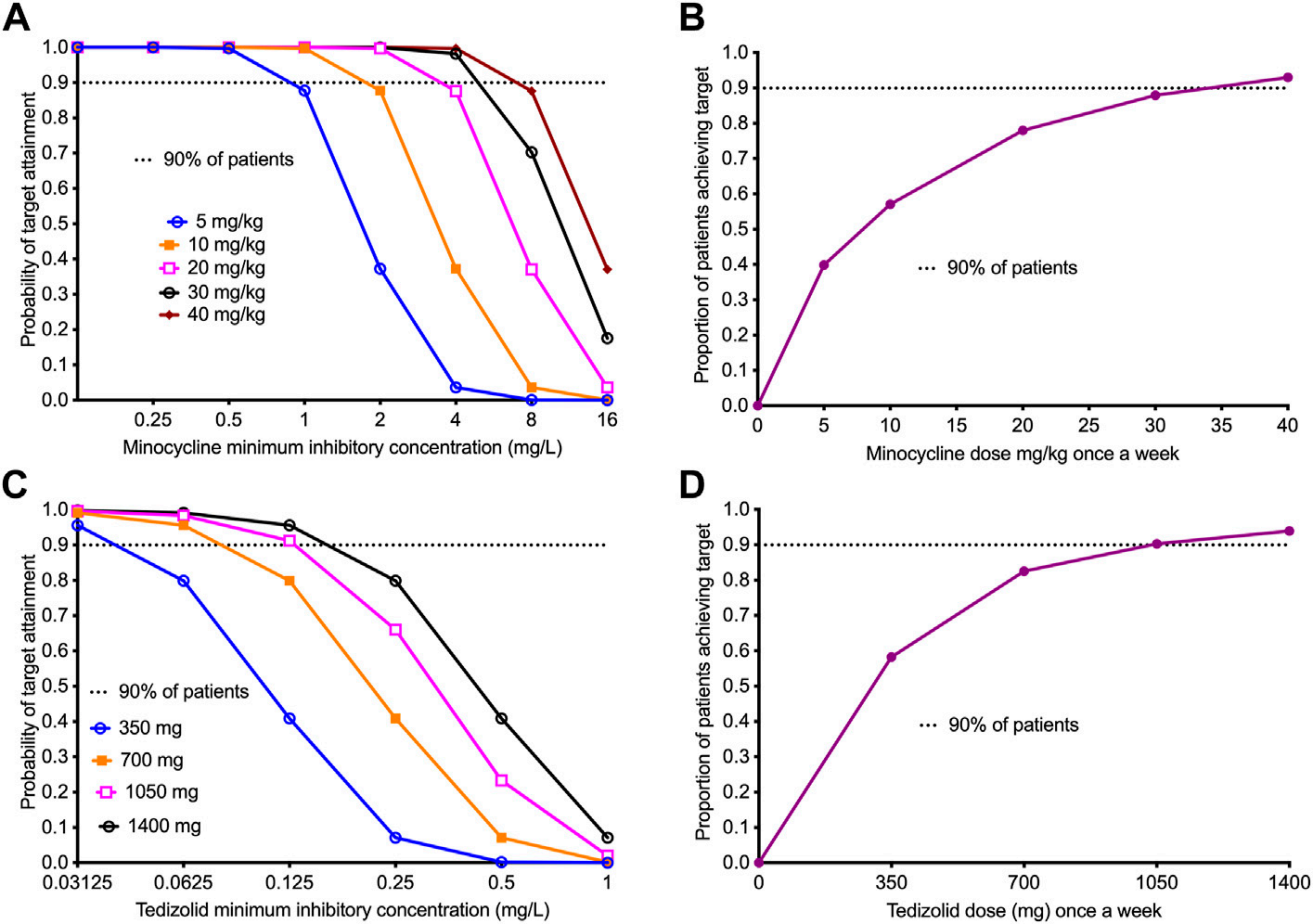
Performance of once-a-week minocycline and tedizolid doses in 10,000 virtual patients with *tuberculosis*. All data points show the proportion of 10,000 patients treated with a dose and dosing schedule who would achieve or exceed the target AUC/MIC exposure associated with optimal effect. 95% confidence intervals were very tight and virtually overlapped with prediction (not shown in the figure). **(A)** The target attainment probability (TAP) for minocycline doses administered once-a-week with the increasing MIC. The TAP of 90% was achieved at doses ≥30 mg/kg up to MIC of 8 mg/L, beyond which the TAP declines indicating that 8 mg/L would be the susceptibility breakpoint for once-a-week dosing. **(B)** The figure shows proportion of 10,000 TB patients achieving the weekly target minocycline cumulative exposure with the different weekly doses. The dose of 30 mg/kg, just shy of the 90% target, was determined as the optimal dose for clinical use. **(C)** The target attainment probability (TAP) for different doses of tedizolid administered weekly with increasing MICs. The TAP with the lowest dose falls below 90% at MIC >0.03125 mg/L, however, for larger doses than 700 mg, the TAP of 90% was achieved up to MIC of 0.125 mg/L. **(D)** The figure shows proportion of 10,000 TB patients achieving the weekly target tedizolid cumulative exposure with the different weekly doses. The dose of 1,050 mg achieved the 90% target and was proposed as the optimal dose.

Next, we performed a 10,000 virtual patient MCE to identify the once-a-week doses of minocycline and tedizolid that would achieve a minocycline AUC_0-168_/MIC of 438 and a tedizolid AUC_0-168_/MIC of 1808.4, for use in patients with TB. We implemented the MCE in ADAPT 5 software: for the domain of input, we utilized the PK parameter estimates and variability of minocycline and tedizolid shown in Table 4 based on several PK studies from the literature and the lung penetration ratios of each drug published elsewhere (Naline et al., 1991; Yamamoto et al., 1999; Gumbo et al., 2004; Fagan et al., 2010; Pasipanodya and Gumbo, 2011). The PK parameter outputs of the 10,000-patient MCE (Table 4) for each drug demonstrate that our simulation experiment accurately recapitulated parameters encountered in the clinic. Figure 5A shows the performance of different once-a-week minocycline doses, starting with 5 mg/kg of minocycline, across a range of minocycline MIC in clinical isolates (Deshpande et al., 2019b). Figure 5B shows that after summation over the MIC distribution, the optimal minocycline dose would be 30 mg/kg once-a-week, which will achieve the target exposure in ∼90% of the patients. For tedizolid, Table 4 also summarizes the PK parameters in the 10,000 simulated TB patients. Figure 5C shows the performance of each once-a-week tedizolid dose over the MIC distribution range reported by Vera-Cabrera et al. Vera-Cabrera et al. (2006). The summation based on that MIC distribution represents the proportion of patients who would achieve the tedizolid exposure in the lung, that was achieved with the once-a-week tedizolid regimen in the HFS-TB (Figure 5D). The tedizolid optimal once-a-week dose was identified as 1,050 mg, which is less than the cumulative amount of 200 mg/day standard dose (i.e, 1,400 mg/week) of that drug for the week.

**TABLE 4:** Pharmacokinetic parameter input and output in Monte Carlo experiments.

	Subroutine PRIOR (clinical studies)		10,000 simulated TB patients	
	Mean	Standard deviation	Mean	Standard deviation
Minocycline				
Clearance (L/hr/kg)	0.03	0.009	0.03	0.01
Volume (L/kg)	0.57	0.26	0.57	0.39
Absorption constant (hr^−1^)	3.00	0.40	3.01	1.14
Tedizolid				
Total clearance (L/hr)	6.69	2.0	6.71	3.71
Central Volume (L)	69	12.42	68.9	29.4
Intercompartmental clearance (L/hr)	0.96	0.29	0.97	0.54
Peripheral volume (L)	13.6	2.44	13.5	5.57
Absorption constant (hr^−1^)	1.99	3.86	1.98	2.85

## Discussion

The present study reports several important findings with regards to the different drug combinations of two- and three-drug regimens for TB. First, we found that daily minocycline dual-drug combinations with rifampin was highly effective and equaled the three-drug regimen of isoniazid-rifampin-pyrazinamide. The combination of minocycline and rifampin is already being employed in the treatment of leprosy and has been shown to be synergistic for the treatment of *Staphylococcus* infections (Zinner et al., 1981; Segreti et al., 1989). In addition, the minocycline derivative omadacycline when combined with rifapentine also demonstrated excellent synergy against *Mycobacterium kansasii* (Singh et al., 2022). Our HFS-TB findings suggest that the minocycline-rifampin combination could also be useful in TB patients, although it will not work with MDR-TB. Moxifloxacin dual therapy was also effective and could be useful in MDR-TB patients, especially if a third drug is added. A potential combination of minocycline-moxifloxacin with the delamanid- OPC-167832 combination which we have noted to be synergistic elsewhere could be explored to shorten therapy duration (Gumbo et al., 2022).

On the other hand, while tedizolid-minocycline had the lowest $\gamma_{f}$ , minocycline, tedizolid, and moxifloxacin, and rifapentine share certain properties that make pairing them with minocycline advantageous. Replacement of rifampin with rifapentine could also take advantage of the remarkable minocycline-rifapentine effect (Singh et al., 2022). First, these anti-microbials agents achieve high *free* drug concentrations in lungs, lung lesions, bone, and cerebrospinal fluid, which are the most common sites of TB (Housman et al., 2012; Dheda et al., 2018; Rifat et al., 2018). Second, their long half-lifes could allow for intermittent dosing for the treatment of TB. Third, the PK/PD driver for these pharmacophores in the treatment of TB is AUC/MIC (Gumbo et al., 2007; Pasipanodya et al., 2013; Chigutsa et al., 2015; Swaminathan et al., 2016; Srivastava et al., 2017; Srivastava et al., 2018b; Deshpande et al., 2018c; Deshpande et al., 2019a; Deshpande et al., 2019b). While the current studies were performed with log-phase growth *Mtb*, elsewhere we have shown that some of these drugs also have excellent sterilizing effect even as intermittent therapy (Srivastava et al., 2018b). Based on the foregoing considerations, if cumulative weekly doses of each drug could be tolerated, combinations would be as effective as once-daily therapy (Srivastava et al., 2018b; Deshpande et al., 2019a; Deshpande et al., 2019b). Indeed, in the case of tedizolid and for all oxazolidinones, the more intermittent the administration the safer it could be, since trough concentrations and time-above certain threshold concentrations have been associated with mitochondrial toxicity (Pea et al., 2012; Flanagan et al., 2015; Song et al., 2015). Addition of a third drug with a long half-life, say OPC-167832 (Hariguchi et al., 2020), could make minocycline-tedizolid-OPC-167832 a highly effective once-a-week regimen with potential to shorten therapy duration. This has major implications for TB programs in the treatment of drug-susceptible, MDR-TB, and XDR-TB. A once-a-week triple drug regimen could dramatically reduce the use of resources by healthcare programs (CDC, 2020).

The possibility of a once-a-week dosing concept led us to ask two related fundamental pharmacology questions. First, can antibiotics continue killing after they are gone from the system? If so, why do such antibiotics continue working after they are gone? Post antibiotic exposure effects are defined as the period of suppression or delay of growth after a short exposure of micro-organisms to antibiotic (Mouton et al., 2005). Common parameters include post antibiotic effect (PAE), sub-MIC effect, post antibiotic sub-MIC effects (PAE SME), which are all are measured as the time (hours or days) it takes to grow 1.0 log_10_ CFU/mL (Mouton et al., 2005). The concept of a PAE is actually as old as the beginning of the antibiotic chemotherapy, though the idea received more systematic impetus when Bill Craig was setting the basis of PK/PD, some 40 years ago (Eagle and Musselman, 1949; McDonald et al., 1977; Vogelman et al., 1988; Craig et al., 1991). Here, we found that long after a bolus of minocycline and the drug was eliminated from the HFS-TB PK system, the drug persisted inside *Mtb* for many days and *continued killing* the bacteria for an additional 5-day, in two separate experiments. Thus, we documented continued *microbial kill* after falling below MIC and detection limits. We propose this as a separate PK/PD parameter from PAEs, post antibiotic microbial killing (PAMK). PAMK is defined as the time (in hours) of continued microbial kill (here quantitively measured as persistently negative γ) after drug falls below the MIC.

The second fundamental question follows from the definition of PAMK: why would such antibiotics continue microbial kill after they are gone? In the case of PAE, the standard explanation has been that antibiotics have a good PAE because of the time it takes for an organism to recover from the effects of an antibiotic and resume normal growth after the brief exposure. Since the most profound PAE was encountered with bacterial protein and nucleic acid synthesis inhibitors, one popular mechanistic explanation has been that inhibition of DNA and protein synthesis upon antibiotic exposure continue for several hours following antibiotic removal, delaying growth, until DNA synthesis resumes at a much later time, also called a “hit-and-run” scenario (Guan et al., 1992; Odenholt et al., 2001; Svensson et al., 2002). However for PAMK, there is persistent microbial kill as if antibiotic is still around. For minocycline the bacterial PKs were consistent with system hysteresis. The concept of hysteresis was first described in electromagnetism by Ewing in 1882 (Ewing, 1882), but mathematical formalism was achieved only recently by Mayergoyz (2003). Hysteresis is when a system (bacterial PKs in this case) lags the input (HFS-TB PK system) but is dependent on that history of input. Here, we found that the PAMK paralleled the system hysteresis. Therefore, we would like to propose the concept of “pharmacologic memory” arising from system hysteresis. By definition, a memory system requires that the dependent variable should retain information at a later time after the input is gone. In basic information theory, Claude Shannon assumed that memory is finite, and that the output would depend on both the history and present state of the system (Shannon, 1997). In our case, the dependent variable is bacterial PK, while the finite input is the extracellular HFS-TB PK system or even the bolus. The minocycline bacterial PK differed from the HFS-TB PK profile, consistent with a dynamic lag and thus system in hysteresis or memory. To our knowledge, this is the first time that bacterial concentrations have been measured when exposed to an external dynamic concentration profile instead of static concentrations: the HFS-TB PKs. On the flip side, it is unclear is PK system hysteresis could have a deleterious effect on the patient. Regarding minocycline adverse events in patients, and the fact that the minocycline concentrations in patient’s circulatory system will decrease below detection for 60% of dosing interval with once-a-week dosing, while persistent in the bacteria, suggests there would be fewer side effects to the patient. However, the effect of bacterial PK system hysteresis on emergence of antimicrobial resistance are unclear, and this will require further urgent study.

There are some limitations to our HFS-TB studies. First, we examined only two laboratory strains in our study. The findings could differ in clinical isolates with varying MICs. Elsewhere we have shown widespread susceptibility of MDR-TB and XDR-TB clinical strains to minocycline and tedizolid (Srivastava et al., 2018b; Deshpande et al., 2019b). Therefore, the effect is not likely limited to laboratory strains only. Second, the tolerability of 30 mg/kg/week of minocycline and 1,050 mg tedizolid that we found to be effective as once-a-week therapy is yet unclear. In the MINOS study with 41 stroke patients who received the highest minocycline dose of 10 mg/kg/day for 3 days (30 mg/kg/week) was well-tolerated and achieved a serum half-life of 24 h (Fagan et al., 2010). Additionally, a recent study demonstrated that there was rapid reversal of tedizolid toxic effects upon discontinuous administration and that an intermittent dosing schedule led to lower tedizolid toxicity (Milosevic et al., 2018). Therefore, a once-a-week regimen is expected to have lower or no toxicity. Nevertheless, these higher doses need to have their safety examined and compared to daily therapy in the clinical setting.

In summary, we found that the best drugs to combine with minocycline for TB were rifampin and moxifloxacin. Second, once-a-week minocycline plus tedizolid regimen was as effective as a daily regimen in the HFS-TB. Third, we measured bacterial PKs in the face of extracellular dynamic PKs and identified system hysteresis. We propose this drug persistence inside *Mtb* and system hysteresis as a basis for a concept of pharmacologic memory. Fourth, we propose the PK/PD concept of PAMK, which could be explained by bacterial PK system hysteresis.

## Data Availability

The original contributions presented in the study are included in the article/Supplementary Material, further inquiries can be directed to the corresponding author.
