# Supplementary figures and images for: Minocycline intra-bacterial pharmacokinetic hysteresis as a basis for pharmacologic memory and a backbone for once-a-week pan-tuberculosis therapy
Source: Front Pharmacol. 2022 Oct 18;13:1024608. doi: 10.3389/fphar.2022.1024608 (PMC9622937; doi:10.3389/fphar.2022.1024608)

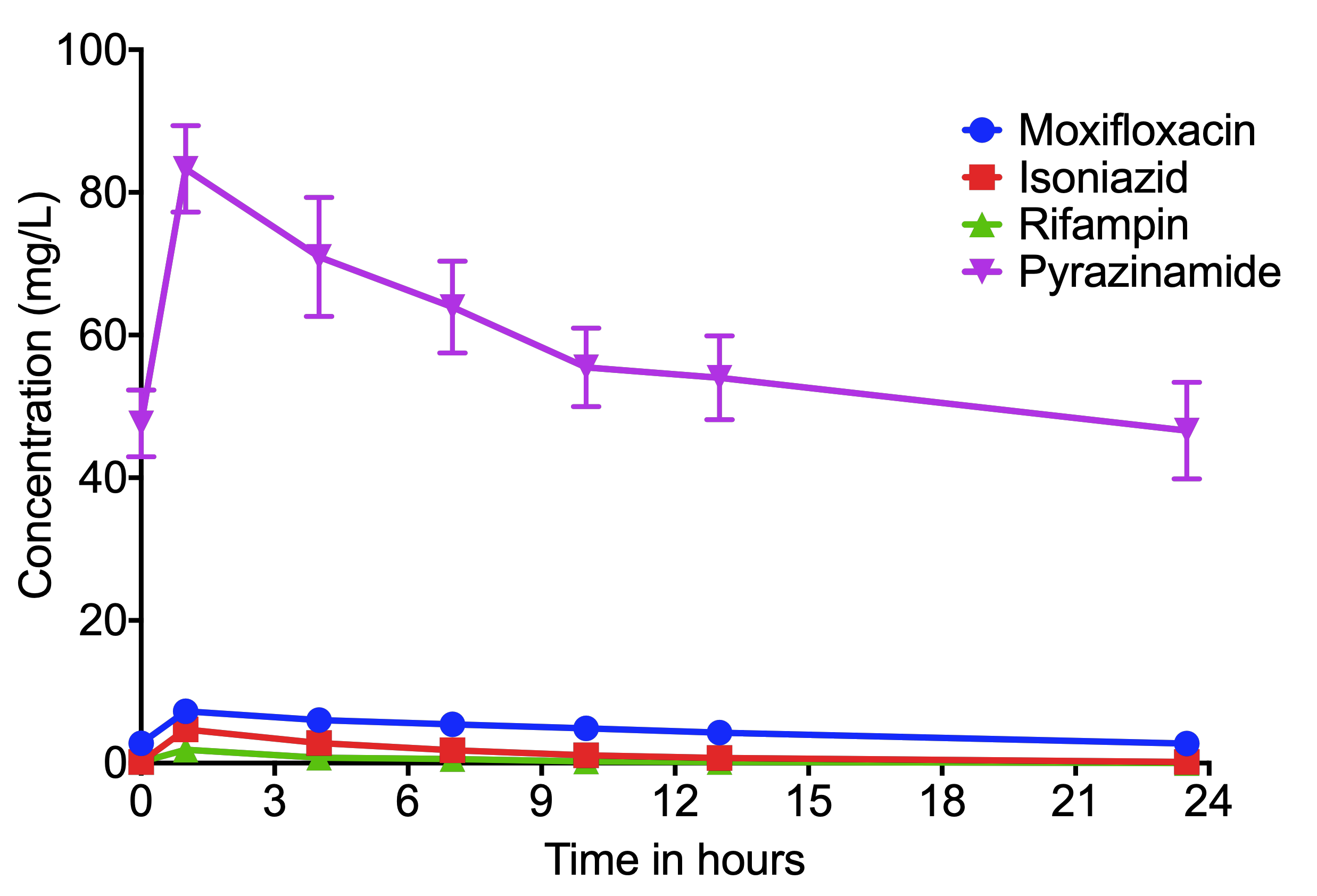

Supplement: Supplementary file 1 [file Image1.TIFF]
